# Effect of Solution Properties in the Development of Cellulose Derivative Nanostructures Processed via Electrospinning

**DOI:** 10.3390/polym14040665

**Published:** 2022-02-10

**Authors:** Pablo Sánchez-Cid, José Fernando Rubio-Valle, Mercedes Jiménez-Rosado, Víctor Pérez-Puyana, Alberto Romero

**Affiliations:** 1 Departamento de Ingeniería Química, Facultad de Química, Universidad de Sevilla, 41012 Sevilla, Spain; vperez11@us.es (V.P.-P.); alromero@us.es (A.R.); 2 Pro2TecS—Chemical Process and Product Technology Research Centre, Department Ingeniería Química, ETSI, Campus de “El Carmen”, Universidad de Huelva, 21071 Huelva, Spain; josefernando.rubio@diq.uhu.es; 3 Departamento de Ingeniería Química, Escuela Politécnica Superior, Universidad de Sevilla, 41011 Sevilla, Spain; mjimenez42@us.es

**Keywords:** electrospinning, cellulose acetate, ethylcellulose, nanostructures, rheological properties, thermal properties, microstructure

## Abstract

In the last few years, electrospinning has proved to be one of the best methods for obtaining membranes of a micro and nanometric fiber size. This method mainly consists in the spinning of a polymeric or biopolymeric solution in solvents, promoted by the difference in the electric field between the needle and collector, which is finally deposited as a conjunction of randomly oriented fibers. The present work focuses on using cellulose derivatives (namely cellulose acetate and ethylcellulose), based on the revaluation of these byproducts and waste products of biorefinery, to produce nanostructured nanofiber through electrospinning with the objective of establishing a relation between the initial solutions and the nanostructures obtained. In this sense, a complete characterization of the biopolymeric solutions (physicochemical and rheological properties) and the resulting nanostructures (microstructural and thermal properties) was carried out. Therefore, solutions with different concentrations (5, 10, 15, and 20 wt%) of the two cellulose derivatives and different solvents with several proportions between them were used to establish their influence on the properties of the resulting nanostructures. The results show that the solutions with 10 wt% in acetic acid/H_2_O and 15 wt% in acetone/*N*,*N*-dimethylformamide of cellulose acetate and 5 wt% of ethylcellulose in acetone/*N*,*N*-dimethylformamide, exhibited the best properties, both in the solution and nanostructure state.

## 1. Introduction

Electrospinning is defined as the spinning of polymeric solutions or molten polymers in the presence of strong electric fields. Thus, this technique allows the fabrication of fibers with diameters between micro and nanometers. The application of a high electric force, enough to overcome the surface tension of the polymeric solution, leads to the generation of fibers from the solution that are finally deposited randomly over the collector, while the solvent evaporates. These fibers move along the direction of the electric field, thus they are unstable and may undergo elongation, depending on the set parameters [1,2]. There are numerous variables that should be considered when designing an electrospinning process, such as compositional parameters (solution concentration, viscosity, conductivity, surface tension, molecular weight of the polymer, etc.), processing parameters (flow rate, voltage, distance between the needle and the collector, etc.), and environmental parameters, such as temperature and relative humidity [1,2,3,4]. In addition, electrospinning is a versatile process that allows obtaining structured nanofibers, which are formed by micro- and nano-sized fibers. Such nanofibrous membranes offer countless advantages over traditional fibers, such as an enormous surface area/volume ratio, surface flexibility, high porosity, porosity control, pore interconnectivity, and superior mechanical performance compared to other known forms of the material. In addition, a wide range of raw materials, such as synthetic polymers, natural polymers, proteins, polysaccharides, etc. can be used to obtain electrospun nanostructures [5]. Due to these characteristics, the use of nanofibers and therefore electrospinning has increased in recent years, making them optimal candidates for a wide variety of applications, including tunable hydrophobicity and water adhesion, scaffolds for tissue engineering, air filtration media, controlled drug release, biosensors, textiles, wound dressings, special membranes, and antimicrobial activities [6,7,8].

Cellulose (C_6_H_10_O_5_)_n_ is the most important and abundant structural polysaccharide and biomolecule in the world [9]. Cellulose was first synthesized in 1992 by Kobayashi and Shoda, without using any enzyme of a biological origin [10,11]. Regarding its structure, cellulose is a glucose polymer formed by β-type glucose molecules attached by β (1→4) glycosidic bonds. Cellulose chains present a linear structure, united by hydrogen bonds. These units are not located exactly in the plane of the structure, but adopt a saddle conformation with the successive glucose residues rotated at an angle of 180° with respect to the molecular axis and the hydroxyl groups in the equatorial position, which provide high mechanical resistance [12,13]. Due to its structure, cellulose is susceptible to considerable modifications to give rise to new derivative compounds that may be more appropriate than cellulose for certain applications [14]. There is a large variety of cellulose derivatives, such as methylcellulose, cellulose acetate, and ethylcellulose, among others, with the latter two being the most widely used [8,14,15,16,17]. These organic compounds are obtained from cellulose, modifying its original structure by substituting external hydroxyl groups with methyl, acetyl, or ethoxy groups, respectively [12].

The electrospinning of cellulose derivatives has been extensively studied in the last decade. However, there have been enormous advances, particularly in the preparation of composite materials based on the electrospinning of these derivatives, which have enormous potential to turn several industrial sectors around [18]. Many researchers have studied electrospun cellulose derivatives nanofibers using different solvent systems [19]. The different solvents used for electrospinning were: Acetone, dimethylacetamide, dimethylformamide, acetic acid, chloroform, methanol, water, or their mixtures in different proportions, with acetone being the most commonly used solvent [20,21,22]. The main problem is that the boiling point (56 °C) of acetone is low, and it evaporates quickly, which hinders the long-term electrospinning required for the large-scale production of nanostructures for various applications.

In recent years, cellulose derivatives electrospun nanostructures have been used as scaffolds for tissue regeneration [23,24], as filter membranes, for catalytic processes, due to their high surface area [25]. Cellulose derivatives fibers have also been utilized in the textile industry, combining different types of fibers, with polymeric coatings [26]. However, the main applications of cellulose derivatives nanostructures are medical applications [27,28], such as the previously mentioned TE, bandage fabrication, drug-controlled release systems, medical implants, and artificial organs [1,12]. Although cellulose derivatives nanostructures potential applications have been investigated, no correlation between the properties of the previous solutions and the properties of electrospun nanostructures has been established yet. Thus, in the present work it is intended to establish a correlation between the physicochemical properties of the solution with the microstructural properties of the electrospun nanostructures, for this purpose it will be evaluated how it affects the concentration of natural and biodegradable polymers, as well as how it affects the solvent used.

Therefore, the main objective of this study was to relate the characteristics of the initial solutions with those of the obtained nanostructures, using cellulose derivatives as raw materials. For this purpose, solutions of ethylcellulose and cellulose acetate were prepared using different weight concentrations as well as different solvents. They were rheological and physically characterized. On the other hand, a complete microstructural, thermal, and chemical characterization of the electrospun fiber mats was carried out. Properties of the initial solution and nanostructures have been correlated to establish relationships between them.

## 2. Materials and Methods

### 2.1. Materials

The polysaccharides chosen in this study were cellulose acetate (AC) (M_w_ = 30,000 g·mol^−1^; acetyl groups percentage = 39.8%; DS = 2.45) and ethylcellulose (EC) (M_w_ = 45,000 g·mol^−1^; ethoxy groups percentage = 48%; DS = 2.45), which are low molecular weight cellulose derivatives. Both materials were provided by SIGMA ALDRICH S.A. (Taufkirchen, Germany). The selected solvents were acetone, provided by Honeywell (Offenbach am Main, Germany), *N*,*N*-dimethyl-formamide (DMF), supplied by EMSURE (Darmstadt, Germany), acetic acid provided by Panreac Química S.A. (Barcelona, Spain), and distilled water. These solvents were chosen based on the great solubility of both cellulose derivatives in them.

### 2.2. Nanofabrication of Cellulose Derivatives

#### 2.2.1. Solution Preparation

AC solutions were prepared by dissolving different concentrations of polymer (5, 10, 15 and 20 wt.%) into a 2:1 mixture of acetone/DMF or acetic acid/water, due to its high solubility in these solvents [14], to evaluate the influence of the solvents in the procedure and the properties of the systems. Each system underwent an agitation step at 500 rpm, using a magnetic stirrer for 4 h.

In the same way, EC was dissolved at different concentrations (2.5, 5, 10, and 15 wt.%) in a 2:1 acetone/DMF solvent, using the same protocol, in order to evaluate the differences between both polymers. It is worth mentioning that acetic acid/water solutions were not prepared for EC due to its low solubility in this solvent [15].

#### 2.2.2. Electrospinning

The fabrication of the nanostructures of AC and EC was developed with a Fluidnatek LE-50, electrospinning equipment (Bioinicia, Valencia, Spain). A total of 5 mL of each solution were introduced into a syringe, equipped with a 21 G needle. The syringe was fixed to the support, using the vertical setting, with a distance of 15 cm from the needle to the collector. The high-voltage power source supplied 17 kV. Each experiment was carried out at room temperature (22 ± 1 °C) and a controlled relative humidity of 45 ± 1%. 

### 2.3. Characterization

#### 2.3.1. Characterization of the Solutions

Once the solutions of each system were prepared, rheological properties, as well as physical properties, such as density, surface tension, and electric conductivity were characterized and analyzed. 

##### Rheological Properties

Rheological measurements were carried out to determine the viscosity of the solutions as a function of the shear rate. The measurements were carried out with a stress-controlled rheometer AR 2000 (TA Instruments, New Castle, DE, USA). In the measurements, the samples were placed into a cone/plate measuring geometry with 1.016° and a diameter of 60 mm, in order to minimize possible inertia effects and to avoid gliding. Each measurement was taken at room temperature and the shear rate range studied was 0.02 to 100 s^−1^. In order to establish a relationship between rheological and microstructural properties, specific viscosity (η_sp_) was determined using Equation (1):

(1) $$\eta_{\mathrm{sp}}=\frac{\eta \;-\;\eta_{0}}{\eta_{0}},$$

where η (Pa·s) is the viscosity of the solution and η_0_ (Pa·s) is the viscosity of the pure solvent.

In addition, extensional viscosity measurements were carried out using a HAAKE CaBER 1 (Thermo Haake GmbH, Germany) at room temperature. One bead of solution was taken between two plates (4 mm of diameter) concentrically set. The plates were separated vertically with a gap of 1 mm and a traction stress was applied to form a fluid unstable filament, observing the evolution of the filament diameter (D_min_) with time, using a micrometer and its interaction with a laser, which provides a way to estimate the extensional viscosity (η_ext_).

##### Physical Properties

Density measurements were carried out using a Densito 30P digital densimeter (Mettler Toledo, Sevilla, Spain). Moreover, surface tension was analyzed using a force tensiometer Sigma 703D (Biolin Scientific, Shanghai, China). The measurements of each system were taken by temporal stability, utilizing a Wilhelmy platinum plate (39.24 mm wide and 0.1 mm thick). Each measurement was recorded at room temperature. In addition, conductivity measurements of the solutions were performed with an EC-Meter BASIC 30+ digital conductometer (Crison Instruments, Barcelona, Spain) by electrical stability.

#### 2.3.2. Characterization of the Nanostructures

##### Chemical Properties

Chemical bonds were analyzed by Fourier-transform infrared spectroscopy (FTIR), using a Hyperion 1000 spectrophotometer (Bruker, Santa Clara, CA, USA). The samples were placed in an ATR diamond sensor to obtain their infrared profile. The measurements were obtained between 4000 and 400 cm^−1^ with an opening of 4 cm^−1^ and an acquisition of 200 scans. Baseline correction was performed by measuring without a sample.

##### Thermal Properties

Thermogravimetric analyses were carried out using TGA Discovery equipment (TA Instruments, USA), evaluating the loss of weight of the systems with temperature, in order to observe the thermal events that may take place. Sample quantities between 4 and 7 mg were placed on platinum pans and heated from 25 °C to 600 °C, at 10 °C·min^−1^, under a N_2_ atmosphere with a flow rate of 60 mL/h. Moreover, differential scanning calorimetry analyses were also carried out, in order to determine the heat flow associated with the different thermal transitions, using a SDT Q600 (TA Instruments, USA). The tests were performed between −50 and 220 °C, with a heating rate of 10 °C·min^−1^ and N_2_ atmosphere with a flow rate of 60 mL/h. AC and EC raw materials were also thermally characterized in order to evaluate if electrospinning process produces changes in the thermal transition of the final nanostructures in respect to the raw material.

##### Microstructural Properties

Prior to microscopy examination, samples (2–3 mm) were cut and treated with osmium vapor (1%) for 8 h to fix the samples and facilitate their observation under the microscope. Then, the fixed samples were covered by a thin film of Au to improve the quality of the micrograph (improving sample conductivity). The microscopy examination was performed using a Zeiss EVO scanning electron microscope (Stuttgart, Germany) with a secondary electron detector at an acceleration voltage of 10 kV. A digital processing free software, FIJI Image-J (National Institutes of Health, Bethesda, MD, USA), was used to determine the pore size distribution and the mean pore size of the nanostructures.

### 2.4. Statistical Analysis

At least three replicates were carried out for each measurement. Statistical analyses were performed with *t*-tests and one-way analysis of variance (*p* < 0.05), using PASW Statistics for Windows (Version18: SPSS Inc., Endicott, NY, USA). Standard deviations were calculated for selected parameters. The significant differences were established with a confidence level of 95% (*p* < 0.05), which are indicated with different letters in the different tables.

## 3. Results

### 3.1. Characterization of the Solutions

As was previously mentioned, the electrospinning process depends on many variables and physicochemical properties of solutions, such as viscosity, surface tension, and electric conductivity. In particular, the viscosity of the solution can be adapted by modifying the polymer concentration [29,30,31] and in fact, some correlations were applied in order to find the minimal concentration that leads to adequate viscosity values to perform the electrospinning process. Table 1 displays the values of shear viscosity of different solutions of AC and EC, as well as other physicochemical parameters, namely, extensional viscosity, density, surface tension, and conductivity. It is important to mention that every system exhibited a Newtonian behavior in the applied shear rate range.

From these results, it can be observed that an increase of the polymer concentration used to make the solution leads to an increase of viscosity, regardless of the solvents used in the process. On the other hand, comparing the results obtained for the AC systems with both combination of solvents, the solvents appear to influence viscosity, obtaining higher viscosities when using acetone/DMF, due to the interaction between the solvents and polymer. Finally, when comparing the effect of the polymer itself, i.e., using different polymers (AC and EC) with the same mixture of solvents, it can be observed that the viscosities of the EC systems are considerably higher, due to the structural properties of each polymer [32]. EC + Acetone/DMF 20 wt.% was not included in Table 1, as it did not provide relevant information, conversely to the 2.5 wt.% solution. The differences in the physicochemical properties of the systems prepared with EC with respect to AC, using in both cases as solvents a DMF/Ac mixture is mainly due to two factors. On the one hand, the main factor is due to the molecular weight, since EC has a molecular weight of 45,000 g.mol^−1^, while CA has a molecular weight of less than 30,000 g.mol^−1^. As is well known, systems made with a polymer with a higher molecular weight have higher viscosities, in addition to causing the displacement towards lower concentrations of the critical concentration (Ce) from which these systems are in the semi-dilute entangled regime and can generate micro and nanofibers during the electrospinning process [31,33].

On the other hand, the viscosity of the systems is affected by the length of the polymer chain. In the case of systems made by EC, the chain of the ethyl functional group is slightly longer than that of systems made with CA (ethoxy group). It is also important to mention that the content of ethyl groups in the raw material from which the systems were prepared is higher than that of acetyl groups (48% of ethyl groups compared to 39.8% of acetyl groups in AC), as can be seen in Section 2.1 [21,22].

**Figure 1:**
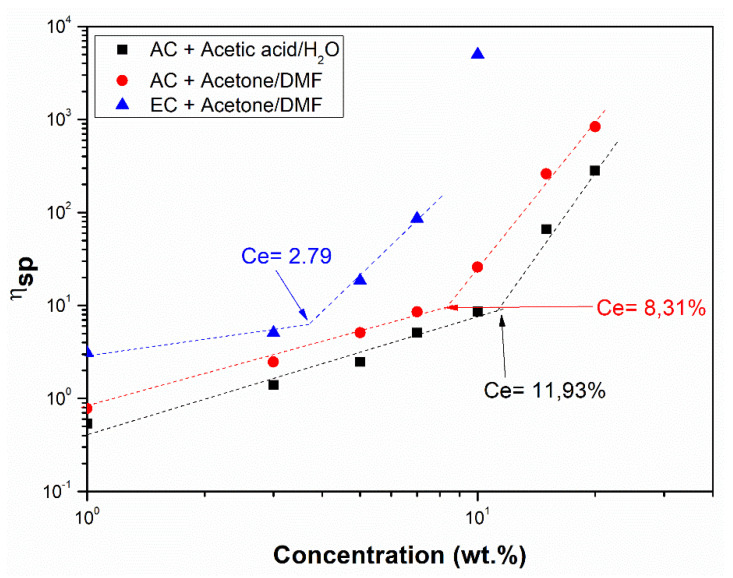
Concentration dependence of the specific viscosity for different solutions of AC and EC with different solvents.

As discussed above, the solution viscosity has often been adapted by modifying the polymer concentration [29,30] and, in fact, some correlations were applied to find the minimum polymer concentration (C_e_) to reach the appropriate viscosity values to perform the electrospinning process. For example, Aslanzadeh et al. [34] determined that a certain viscosity threshold corresponding to the critical polymer overlap concentration is required to obtain relatively uniform nanofibers. To obtain this C_e_, specific viscosity η_sp_ was plotted vs. polymer concentration (Figure 1). 

Figure 1 shows the relationship between the specific viscosity (η_sp_) and derivates celluloses concentrations. The critical entanglement concentration (C_e_) delimiting the semi-diluted unentangled and the semi-diluted entangled regimes can be obtained as the change in the slope of this plot. It must be highlighted that C_e_ strongly depends on the polymer and the solvents used. On the semi-dilute unentangled, strong polymer-solvent interactions predominate, which means that in this regime, no electrospun fibers would be obtained; whereas at higher concentrations (C > C_e_), the predominant process would be electrospinning, thus obtaining nanofibers [29,30].

In addition to rotational viscosity, Table 1 displays the values of extensional viscosity, density, surface tension, and conductivity obtained for each system. Extensional viscosity (η_ext_) reflects the same behavior as the one shown by rotational viscosity, increasing with polymer concentration regardless of the solvent mixture used. Moreover, when comparing each system, similar results are observed, obtaining higher extensional viscosity values for AC when using acetone/DMF as solvents; with the EC systems exerting higher values than the AC systems. Density values are determined by the combination of solvents used, obtaining higher values for AC + acetic acid/H_2_O and similar values for systems with different polymers, however the same solvents. Regardless of the solvents used, increasing the polymer concentration slightly increased density values [35,36]. On the other hand, surface tension results did not show a clear deviation pattern for the studied systems, with the ones obtained for the EC being slightly higher. Finally, conductivity values generally decreased when the polymer concentration was increased, except for the case of the low polymer concentration AC + acetic acid/H_2_O solutions. This could be due to the fact that, when increasing the polymer concentration in the solutions, the number of electric charges also increases up to a maximum concentration, from which, despite the increase in the number of charges, conductivity decreases due to the fact that the movement of the charges becomes progressively more limited [29,30].

### 3.2. Characterization of the Nanostructures

#### 3.2.1. Chemical Properties

**Figure 2:**
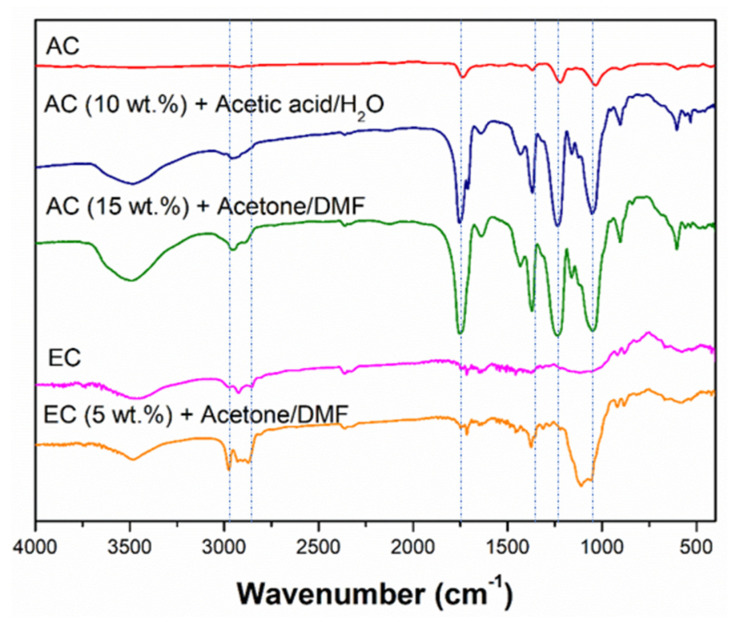
FTIR spectra of both raw materials (AC and EC) and selected nanostructures of the different systems. The dashed lines marked the most representative peaks that have been analyzed.

Figure 2 shows the resulting FTIR spectra of both raw materials (AC and EC) without electrospinning and a representative nanostructure processed with each solvent, namely AC (10 wt.%) + acetic acid/H_2_O, AC (15 wt.%) + acetone/DMF and EC (5 wt.%) + acetone/DMF. These systems are selected as representative because they are the concentrations of each biopolymer immediate in each case to the overcoming of the threshold of the critical concentration (C_e_), from which each system is expected to present microstructural properties formed by micro and nanofibers, being able to speak already of nanostructures that are composed by fibers.

The AC spectrum shows the characteristic bands of esters at 1735 cm^−1^, due to the existence of double bonds conjugated with the carbonyl group, at 1300 cm^−1^ and 1000 cm^−1^. These bands can also be observed in the AC nanostructures, both using acetic acid/H_2_O and acetone/DMF solvents combinations. The differences with the raw AC spectrum are the intensity of the common bands, which are higher for the nanostructures, with respect to the raw material, and the presence of the characteristic bands of the solvents used, namely the ones of carboxylic acids in the case of using acetic acid and water as solvents or the characteristic bands of ketones and amides when using the acetone/DMF mixture. On the other hand, in the case of EC systems, characteristic bands of ethers, ketones, and amides (2950–2800 cm^−1^) can be observed, due to the molecular structure of the polymer itself and the solvents used [37,38,39,40,41,42]. The same increase in the common bands can be observed in this case.

#### 3.2.2. Thermal Properties

Table 2 displays the values of onset temperatures (T_onset_), maximum temperatures (T_max_), weight loss, and residues of the thermal events observed on each processed nanostructure and in both raw materials, as well as the glass transition temperatures (T_g_). The thermal profile of TGA and DSC of the different systems are incorporated as supplementary material (Figures S2 and S3). It is worth mentioning that AC (5 wt.%) + acetone/DMF and EC (2.5 wt.%) + acetone/DMF were not included in Table 2, since it was not possible to carry out TGA/DSC assays, due to the fact that neither of the nanostructures could be removed appropriately. This was due to the fact that the nanostructures could not be correctly separated from the aluminum foil enveloping the aluminum collecting plate. This is an experimental limitation since the structure of these two systems consisted entirely of micrometer-sized particles, which formed randomly arranged agglomerates, and when attempting to remove these nanostructures, part of the aluminum foil was always extracted, which could distort the thermal measurements.

On the one hand, it must be highlighted that both T_onset_ and T_max_ columns include two values, which correspond to two different thermal events (Figure S1). The first one appears at temperatures of 250–300 °C and corresponds to the degradation of acetyl and ethoxyl groups of AC and EC, respectively. The other one occurs at about 350 °C and corresponds to the degradation of cellulose [35,36]. Thus, the first values of these two columns, the T_onset_ and T_max_, of the first thermal event and the second ones correspond to values obtained for the second thermal event.

On the other hand, it can be noted that an increase of polymer concentration causes a slight increment of both T_onset_ and T_max_. Moreover, this increment of polymer concentration also provokes a higher generation of residues.

Furthermore, comparing the effect of the solvents on the different AC systems, it can be observed that, using acetic acid/water as a solvent, higher T_onset_, T_max_, and residue values were obtained with respect to the values obtained for the acetone/DMF combination (Figures S2 and S3). A similar behavior was observed when analyzing the effect of the polymer, obtaining similar values for the AC and EC systems and the same tendency for T_onset_. Additionally, it can be noted that the residue percentages are lower for the processed nanostructures than for the raw materials. This effect could be due to the fact that the electrospinning process enables the formation of fibers with the polymer without dragging the inorganic impurities (i.e., salts) of the raw materials The tendencies observed for the EC systems were similar to those of the AC systems.

Moreover, the T_g_ values of the processed nanostructures were generally higher than the ones obtained for the raw materials, possibly due to the higher crosslinking degree, as the new nanostructure is formed by an entangled nanofiber, whose movement is more limited. Only selected nanostructures had their T_g_ measured, which were AC (10 wt.%) + acetic acid/H_2_O, AC (15 wt.%) + acetone/DMF, and EC (5 wt.%) + acetone/DMF, since although there were no significant differences with the other nanostructures of each system, the ones selected had comparatively better properties than the rest.

#### 3.2.3. Microstructural Properties

**Figure 3:**
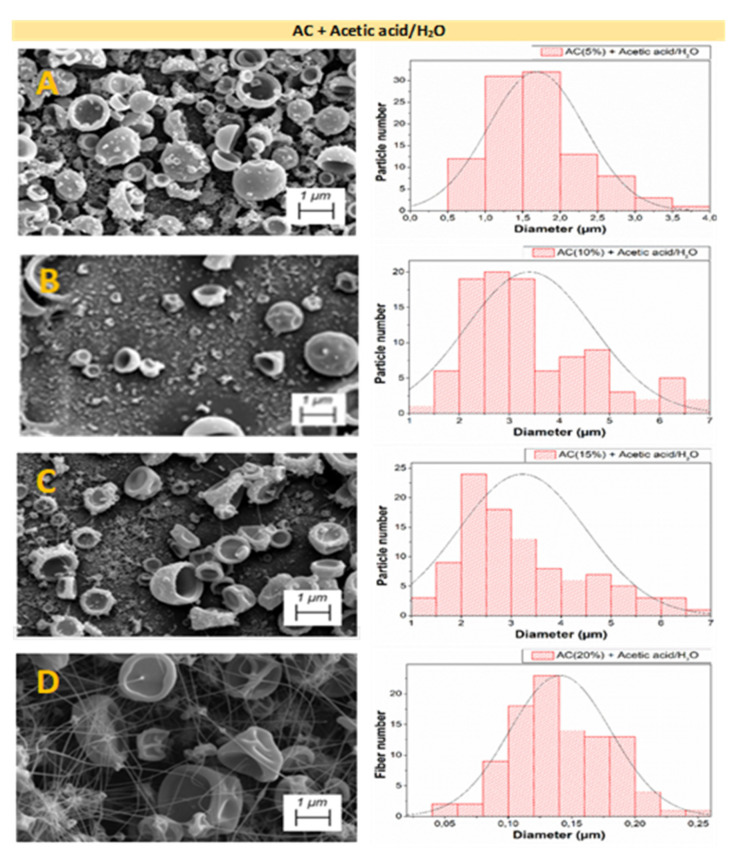
SEM images and distribution of microstructural properties of the nanostructures made with different AC concentrations: (**A**) 5 wt.%, (**B**) 10 wt.%, (**C**) 15 wt.%, and (**D**) 20 wt.%, using acetic acid and water as solvents.

**Figure 4:**
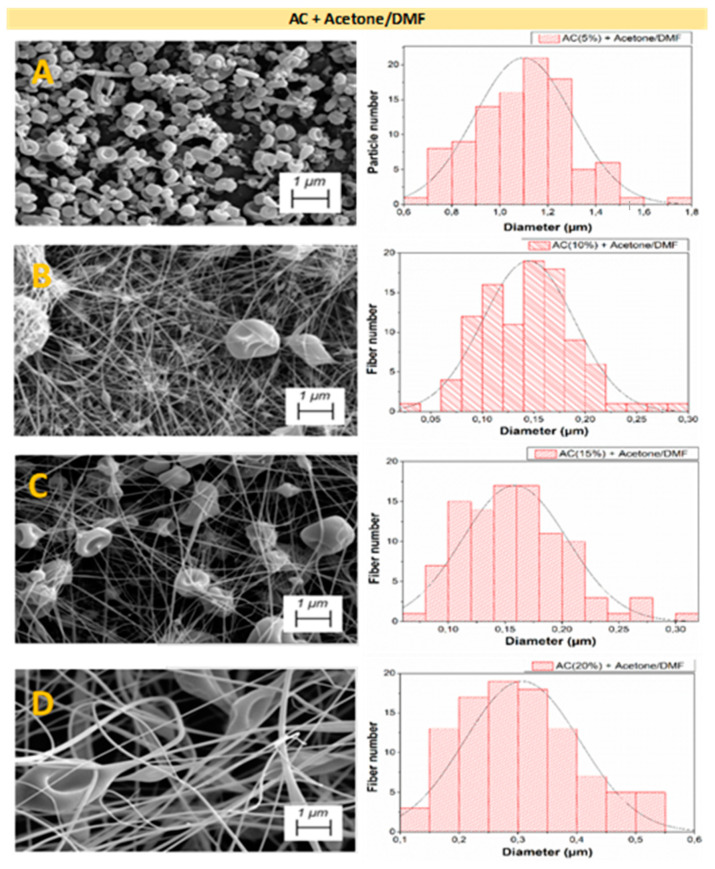
SEM images and distribution of microstructural properties of the nanostructures made with different AC concentrations: (**A**) 5 wt.%, (**B**) 10 wt.%, (**C**) 15 wt.%, and (**D**) 20 wt.%, using acetone and DMF as solvents.

**Figure 5:**
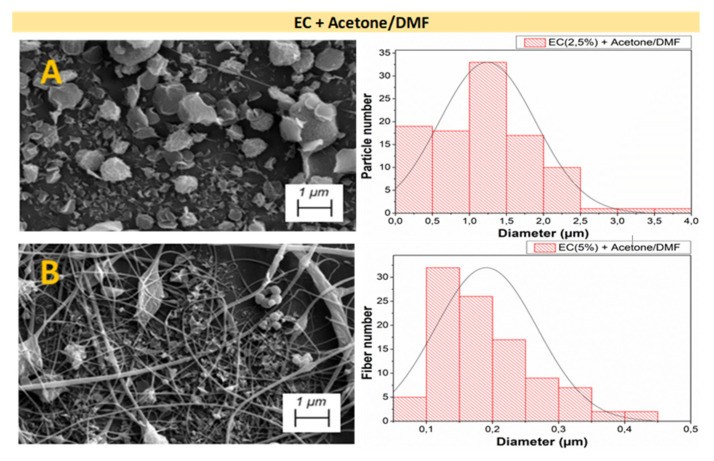
SEM images and distribution of microstructural properties of the nanostructures made with different EC concentrations (**A**) 2.5 wt.% and (**B**) 5 wt.%, using acetone and DMF as solvents.

Figure 3, Figure 4 and Figure 5 shows the analysis of the microstructural properties of every different nanostructured system, displaying both SEM image and the distribution of the microstructural properties for AC + Acetic acid/H_2_O, AC + Acetone/DMF, and EC + Acetone/DMF systems, respectively.

Figure 3A–C shows an increase in the size of nanoparticles when increasing polymer concentration, which means that these results are in line with the obtained rheological properties of the solutions of each system, as the systems in Figure 3A,B were below C_e_ and consequently, as expected, no fibers were obtained in these systems. Conversely, Figure 3C is slightly above C_e_, which is why, in these systems, both particles and fibers with very small diameters were obtained in these systems. Finally, Figure 3D shows a distribution of interconnected particles and fibers, with the latter being larger than the fibers obtained in Figure 3C.

Figure 4 shows the results of microstructural analyses carried out for the AC systems using acetone/DMF as solvents. Once again, the obtained results are consistent with the previous rheological characterization of the solutions, as only particles can be observed in Figure 4A, since this concentration of AC with this combination of solvents is lower than C_e_, whereas Figure 4B–D, whose concentrations are in the regime predominated by the electrospinning, shows predominates and fibers with an increasing size as the polymer concentration is increased.

When comparing the influence of the selected solvents for AC systems, it can be observed that, in solutions with acetic acid/H_2_O, particle formation is favored rather than fiber spinning, due to the difficulty that the solution presented for the electrospinning process. Conversely, AC solutions with acetone/DMF achieved a more abundant fiber formation with irregular morphology. This is due to the difference in the physicochemical properties of the solvents used, which leads to different interactions with CA, in this case, therefore obtaining different physicochemical properties for the resulting solutions, which explains the difference in the C_e_ when using different solvents [43,44]. This fact bears out the importance of making a previous rheological characterization in order to obtain C_e_ and to determine both regimes.

Figure 5 shows the results of the microstructural characterization results of the EC systems. In Figure 5A, only irregular particles can be observed, without fiber formation. Nevertheless, in Figure 5B, when C_e_ was exceeded, a heterogeneous distribution of fibers was obtained. The rest of the EC systems were not included in this analysis due to the fact that it was not possible of electrospun solutions with higher concentrations, since maintaining the electrospinning parameters used in the other systems, resulted in the high viscosity of the solutions made the formation of the Taylor cone impossible [45].

By comparing both raw materials (AC and EC) using the same solvents, it can be concluded that, for the same polymer concentration, fibers and particles with larger diameters were obtained for the EC systems (Table 3), highlighting the influence that the selected polymer exerts on both the rheological properties of the solutions and the properties of the final nanostructures. Moreover, the values of Table 3 corroborate those results observed in diameter distribution for every system in Figure 3, Figure 4 and Figure 5, that is the increment of particle and fiber diameter when increasing the polymer concentration. 

**Figure 6:**
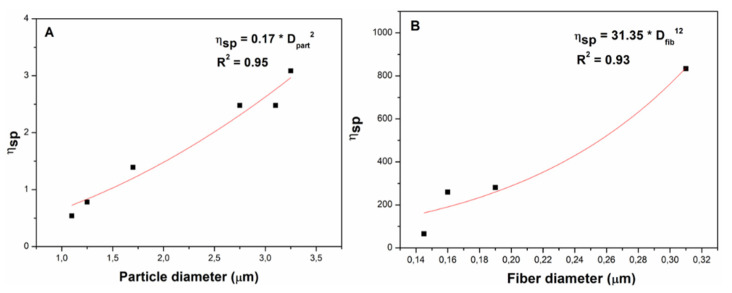
Empirical correlation between specific viscosity of solutions with (**A**) particle diameter and (**B**) fiber diameter obtained for the AC and EC systems.

From the particle and fiber diameters of the different systems, it is possible to correlate the specific viscosity of electrospun solutions with the mean diameter of both particles and fibers (Figure 6). This correlation can be established from specific viscosities in the semi-diluted unentangled regimes for particles (in other words, specific viscosities from concentrations below C_e_) (Figure 6A) and specific viscosities of solutions with concentrations above C_e_ for fibers (Figure 6B). These correlations allow one to determine the values of specific viscosity required to obtain certain diameters of particles or fibers.

## 4. Conclusions

As a general conclusion of the study, a relationship between the initial solutions and final nanostructures was established. It was corroborated that the chemical, thermal, and microstructural properties (fiber size, particle size, and porosity) of the different cellulose derivatives strongly depended on the physicochemical and rheological properties of the solutions, which are provided for the different molecular weight of the raw materials and the solvent used. 

The results obtained show that solutions could be prepared with different cellulose derivatives at different concentrations and different solvent ratios. In general, the solutions of AC at 10 wt.% in acetic acid/H_2_O and 15 wt.% in acetone/DMF, and those of EC at 5 wt.% in acetone/DMF are the ones that present the best compositional and final properties of the nanostructures obtained. In addition, the processing parameters established in the electrospinning allow for obtaining AC nanostructures without difficulties in the process. However, the EC solutions generated problems for electrospinning at higher concentrations (10, 15, and 20 wt.%) whose viscosity values were too high to be processed using the selected electrospinning parameters. Furthermore, an empirical relationship could be established between particle and fiber diameters and the specific viscosity of solutions, which allowed for determining the required value of specific viscosity (and, therefore, polymer concentration) to obtain a certain particle or fiber size. Thus, these results open the possibility of estimating the properties obtained in the nanofibers processed by electrospinning through the characterization of the biopolymeric solutions. However, future studies will characterize the nanostructures in depth to evaluate their functionality.

## Figures and Tables

**Table 1:** Rotational viscosity (η), extensional viscosity (η_ext_), density (ρ), surface tension (γ), and conductivity (σ) values of the different AC and EC solutions. Different letters in each column present significant differences between the parameters (*p* < 0.05).

Systems		η (Pa·s)	η_ext_ (Pa·s)	ρ (g·cm^−3^)	γ (mN·m^−1^)	σ (μS·cm^−1^)
AC + Acetic acid/H_2_O (2:1)	5 wt.%	0.004 ^a^	0.011 ^A^	1.067 ^α^	29.75 ^I^	184.0 ^a^
	10 wt.%	0.011 ^a^	0.035 ^B^	1.074 ^α^	30.15 ^II^	197.7 ^b^
	15 wt.%	0.185 ^b^	0.575 ^C^	1.087 ^β^	31.25 ^III^	174.3 ^c^
	20 wt.%	0.311 ^c^	0.983 ^D^	1.095 ^β^	30.75 ^II^	157.7 ^d^
AC + Acetone/DMF (2:1)	5 wt.%	0.006 ^a^	0.015 ^A^	0.862 ^γ^	30.35 ^II^	128.1 ^e^
	10 wt.%	0.105 ^d^	0.335 ^E^	0.881 ^δ^	31.54 ^III^	113.1 ^f^
	15 wt.%	0.385 ^e^	1.105 ^F^	0.892 ^δ^	32.15 ^IV^	96.71 ^g^
	20 wt.%	0.523 ^f^	1.529 ^G^	0.902 ^ε^	31.74 ^III^	73.13 ^h^
EC + Acetone/DMF (2:1)	2.5 wt.%	0.009 ^a^	0.029 ^B^	0.859 ^γ^	30.58 ^II^	144.5 ^i^
	5 wt.%	0.132 ^g^	0.356 ^H^	0.869 ^γ^	32.51 ^IV^	97.57 ^g^
	10 wt.%	0.678 ^h^	2.104 ^I^	0.877 ^γ^	33.58 ^V^	69.27 ^h^
	15 wt.%	1.115 ^i^	3.395 ^J^	0.886 ^δ^	33.47 ^V^	25.20 ^j^

**Table 2:** Onset temperature (T_onset_), maximum temperature (T_max_), weight loss, residue percentage, and glass transition temperature (T_g_) values obtained for the different raw materials and nanostructured systems of AC and EC.

Systems		T_onset_ (°C)	T_max_ (°C)	Weight Loss (%)	Residue (%)	T_g_ (°C)
AC		272.0/345.4	285.6/367.9	81.9	18.1	171.1
AC + Acetic acid/H_2_O (2:1)	5 wt.%	258.7/330.8	305.8/356.7	88.7	11.3	--
	10 wt.%	259.9/335.3	307.2/358.8	88.1	11.9	190.1
	15 wt.%	260.8/337.4	309.9/358.8	87.5	12.5	--
	20 wt.%	261.6/337.7	311.9/359.8	87.3	12.7	--
AC + Acetone/DMF (2:1)	10 wt.%	257.2/322.8	314.4/356.8	87.9	12.1	--
	15 wt.%	259.0/329.0	314.8/357.2	87.8	12.2	188.2
	20 wt.%	260.0/334.7	315.4/359.3	87.5	12.5	--
EC		261.2/322.9	283.1/354.2	95.2	4.8	181.5
EC + Acetone/DMF (2:1)	5 wt.%	253.1/337.2	308.3/359.8	95.5	4.5	183.0

**Table 3:** Mean values of particles diameter, fiber diameter, and porosity obtained for the different AC and EC systems. Different letters in each column represent significant differences between the parameters (*p* < 0.05).

Systems		Particle Diameter (μm)	Fiber Diameter (μm)	Porosity (%)
AC + Acetic acid/H_2_O (2:1)	5 wt.%	1.70 ± 0.12 ^a^	--	24.29 ± 0.18 ^α^
	10 wt.%	3.40 ± 0.09 ^b^	--	22.72 ± 0.16 ^β^
	15 wt.%	3.05 ± 0.14 ^b^	0.06 ± 0.01	28.19 ± 0.27 ^γ^
	20 wt.%	5.00 ^c^ ± 0.27	0.14 ± 0.01 ^A^	25.30 ± 0.25 ^α,γ^
AC + Acetone/DMF (2:1)	5 wt.%	1.10 ± 0.04 ^d^	--	30.42 ± 0.28 ^δ^
	10 wt.%	3.10 ± 0.11 ^e^	0.15 ± 0.01 ^A^	26.65 ± 0.15 ^γ^
	15 wt.%	3.90 ± 0.19 ^f^	0.16 ± 0.01 ^A^	33.32 ± 0.38 ^ε^
	20 wt.%	--	0.31 ± 0.03 ^B^	25.67 ± 0.41 ^γ^
EC + Acetone/DMF (2:1)	2.5 wt.%	1.25 ± 0.03 ^d^	--	35.45 ± 0.21 ^ζ^
	5 wt.%	2.75 ± 0.08 ^g^	0.19 ± 0.03 ^C^	26.21 ± 0.26 ^γ^

## Data Availability

The data presented in this study are available on request from the corresponding author.

## Supplementary Materials

The following supporting information can be downloaded at: https://www.mdpi.com/article/10.3390/polym14040665/s1. Figure S1: (A) TGA profile and (B) DSC analysis of EC (5 %) + acetone/DMF membrane as representative to show the selected parameters. (1) and (2) refers to the thermal events that occurs during the heating process, namely the degradation of acetyl and ethoxy groups of AC and EC, respectively (1) and the degradation of cellulose (2); Figure S2: TGA analysis of electrospun nanostructures for systems made with: A) AC + Acetic acid/H2O, B) AC + Acetone/DMF and C) EC + acetone/DMF; Figure S3: DSC analysis of AC, AC (10 wt.%) + Acetic acid/H2O, AC (15 wt.%) + Acetone /DMF, EC and EC (5 %) + acetone/DMF.
