# Supplementary material for: Effect of Solution Properties in the Development of Cellulose Derivative Nanostructures Processed via Electrospinning
Source: Polymers (Basel). 2022 Feb 10;14(4):665. doi: 10.3390/polym14040665 (PMC8874405; doi:10.3390/polym14040665)
Supplement: Supplementary file 1 [file polymers-14-00665-s001.zip › polymers-1560768-supplementary.pdf]

SUPLEMENTARY MATERIAL

Cellulose derivatives nanostructures processed via electrospinning

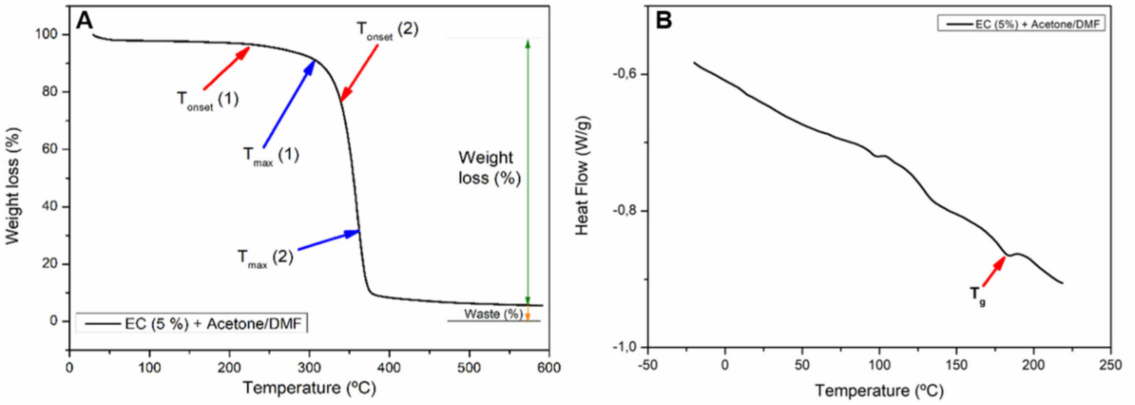

Figure S1. (**A**) TGA profile and (**B**) DSC analysis of EC (5 %) + acetone/DMF membrane as representative to show the selected parameters. (1) and (2) refers to the thermal events that occurs during the heating process, namely the degradation of acetyl and ethoxy groups of AC and EC, respectively (1) and the degradation of cellulose (2).

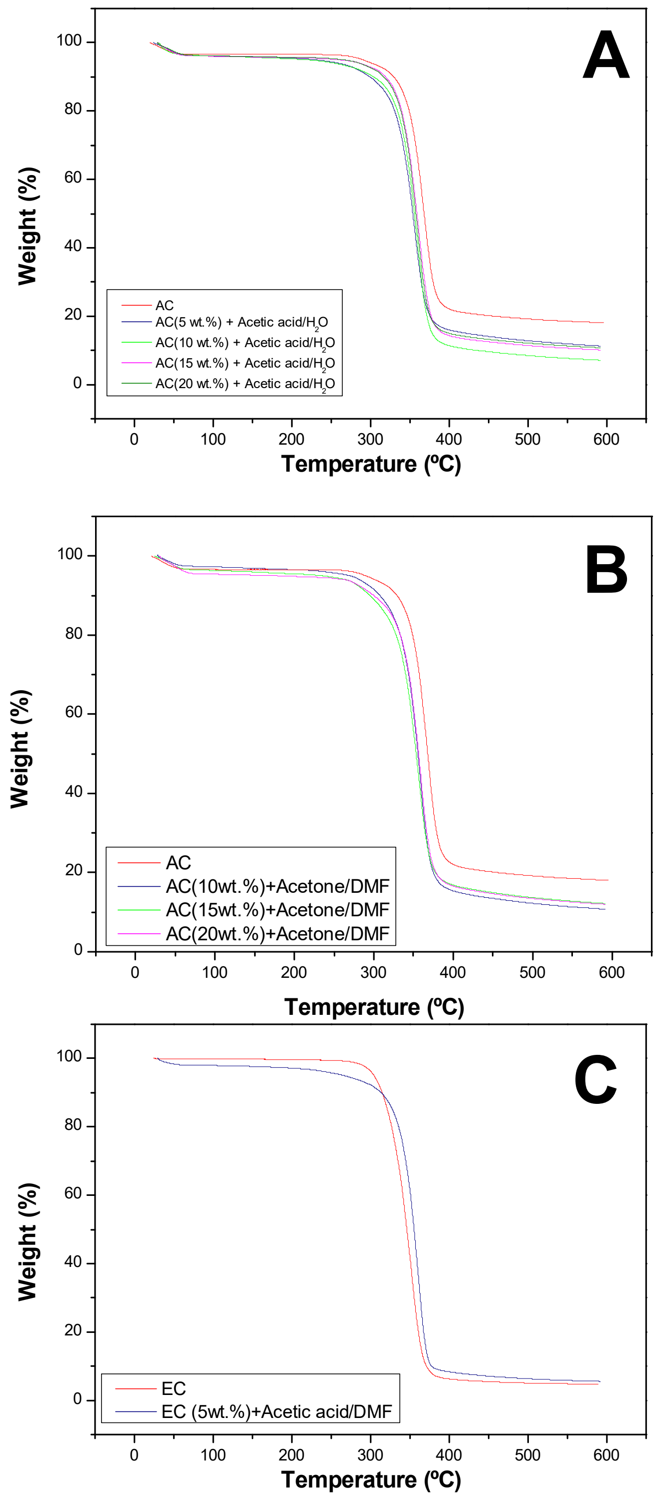

Figure S2. TGA analysis of electrospun nanostructures for systems made with: (**A**) AC + Acetic acid/H_2O, (**B**) AC + Acetone/DMF and (**C**) EC + acetone/DMF.

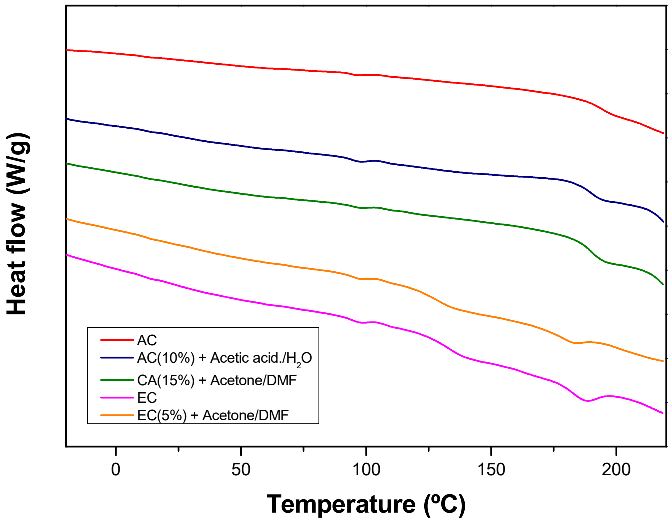

Figure S3. DSC analysis of AC, AC (10 wt.%) + Acetic acid/H_2O, AC (15 wt.%) + Acetone /DMF, EC and EC (5 %) + acetone/DMF.
